# Mapping the Natural History of Benign DICER1-Related Lesions and Identifying Predictors of Malignancy

**DOI:** 10.26502/fjhs.407

**Published:** 2026-04-03

**Authors:** Varsha Karthikeyan, Devendra K Agrawal

**Affiliations:** Department of Translational Research, College of Osteopathic Medicine of the Pacific, Western University of Health Sciences, Pomona, California 91766 USA

**Keywords:** Argonaute strand switch, Ciliary body medulloepitheliomas, Cystic nephroma, DICER1 syndrome, Differentiated thyroid carcinoma, Malignant progression, MicroRNA biogenesis, 3p-strand bias, Pleuropulmonary blastoma, RNase IIIb domain, Sertoli-Leydig cell tumor, Surveillance Guidelines, Tumor predisposition syndrome, Vision Transformer-based radiography

## Abstract

DICER1 syndrome is a complex autosomal dominant tumor predisposition disorder characterized by a distinct chronological progression of benign and malignant lesions. By mapping the transition from early-childhood pulmonary and renal manifestations to the adolescent emergence of endocrine and reproductive neoplasms, this review provides a longitudinal framework for clinical vigilance. Central to this analysis is the molecular “two-hit” mechanism, specifically investigating how somatic hotspot mutations in the RNase IIIb domain disrupt the miR-140/FGF9 signaling axis and the *let-7* feedback loop. The review identifies the neomorphic “Argonaute strand switch” as a primary driver of pathogenesis, resulting in a diagnostic 3p-strand bias that fuels sarcomatous transformation. Beyond the molecular substrate, we define the critical radiographic and clinical markers of malignancy, such as rapid volumetric growth, cystic solidification, and the detection of somatic hotspots via high-sensitivity droplet digital PCR. By integrating the 2024 international surveillance standards with emerging technologies, including Vision Transformer-based radiographic analysis and circulating tumor DNA monitoring, this review offers a proactive, evidence-based roadmap for identifying the predictors of malignancy and better management of the disease. Ultimately, this synthesis aims to equip clinicians and other healthcare profesionals with the predictive tools necessary to achieve definitive cures while minimizing the cumulative clinical and psychological burden on this genetically vulnerable population.

## Introduction

The discovery of DICER1 syndrome has fundamentally shifted our understanding of how microRNA (miRNA) dysregulation fuels human oncogenesis [1, 2]. As an autosomal dominant tumor-predisposition disorder, it presents a strikingly diverse clinical spectrum. This pleiotropic nature often manifests as rare embryonal tumors in early childhood, followed by a shift toward endocrine and reproductive neoplasms in adolescence and adulthood [1, 3]. Recent genome-first approaches in population-scale cohorts have further clarified this prevalence, noting that while the clinical spectrum is broad, many carriers remain asymptomatic or present with benign phenotypes like multinodular goiter [4]. At its core, the syndrome stems from germline pathogenic variants in the DICER1 gene (chromosome 14q32.13), which encodes an RNase III endonuclease essential for the miRNA biogenesis pathway [1, 5, 6, 7]. By precisely processing precursor miRNAs into functional molecules, the DICER1 protein maintains the post-transcriptional brakes on gene networks that govern cellular homeostasis, organogenesis, and stem cell maintenance [2, 8]. Functioning as a molecular ruler, the DICER1 protein relies on the coordinated action of its PAZ, platform, and RNase III catalytic domains to ensure the precision of RNA interference [6, 9]. When this machinery is compromised, typically via a “two-hit” mechanism involving a germline mutation and a subsequent somatic hotspot mutation, the resulting biochemical failure triggers profound developmental errors [2, 10]. Despite the severity of the potential malignancies, the syndrome is marked by incomplete penetrance and significant variable expressivity [1, 3, 4].

For the clinician, this clinical uncertainty necessitates a deep characterization of the natural history of benign lesions. The ability to differentiate indolent cysts from emerging sarcomas is the primary factor in determining patient management [1, 11]. Rare mosaic missense mutations in the RNase IIIb domain can also lead to distinct phenotypes such as GLOW syndrome (Global developmental delay, Lung cysts, Overgrowth, and Wilms tumor), expanding the known clinical reach of DICER1 [12]. By identifying the clinical, radiological, and molecular markers of this malignant shift, we can transition from reactive management to a proactive, surveillance-based model [1, 11, 13].

### Natural History of Benign DICER1-Associated Lesions

#### Pulmonary and Renal Manifestations in Early Childhood

The natural history of DICER1-associated lesions follows a distinct chronological clock, with pulmonary and renal manifestations typically appearing within the first five years of life [1, 11]. Pleuropulmonary blastoma (PPB) is the most frequent sentinel event [14]. It often originates as Type I PPB, air-filled, multicystic structures that are easily misidentified on initial imaging as simple congenital pulmonary airway malformations [11, 15]. Pathologically, while these cysts are lined by benign respiratory epithelium, they are characterized by a subepithelial cambium layer of primitive rhabdomyoblastic cells [11, 15, 16]. Interestingly, a subset of these lesions may undergo regression (Type Ir), where the primitive cells disappear and leave behind stable, non-neoplastic cysts [11, 17]. However, the risk of cystic Type I lesions progressing into solid Type II or III sarcomas remains a critical concern during this early developmental window [1, 11, 17]. Rare variants of PPB-like manifestations, such as thoracic Sertoli-Leydig cell tumors or PPB-like peritoneal sarcomas, further illustrate the pleiotropic nature of the syndrome [18, 19].

**Figure 1:**
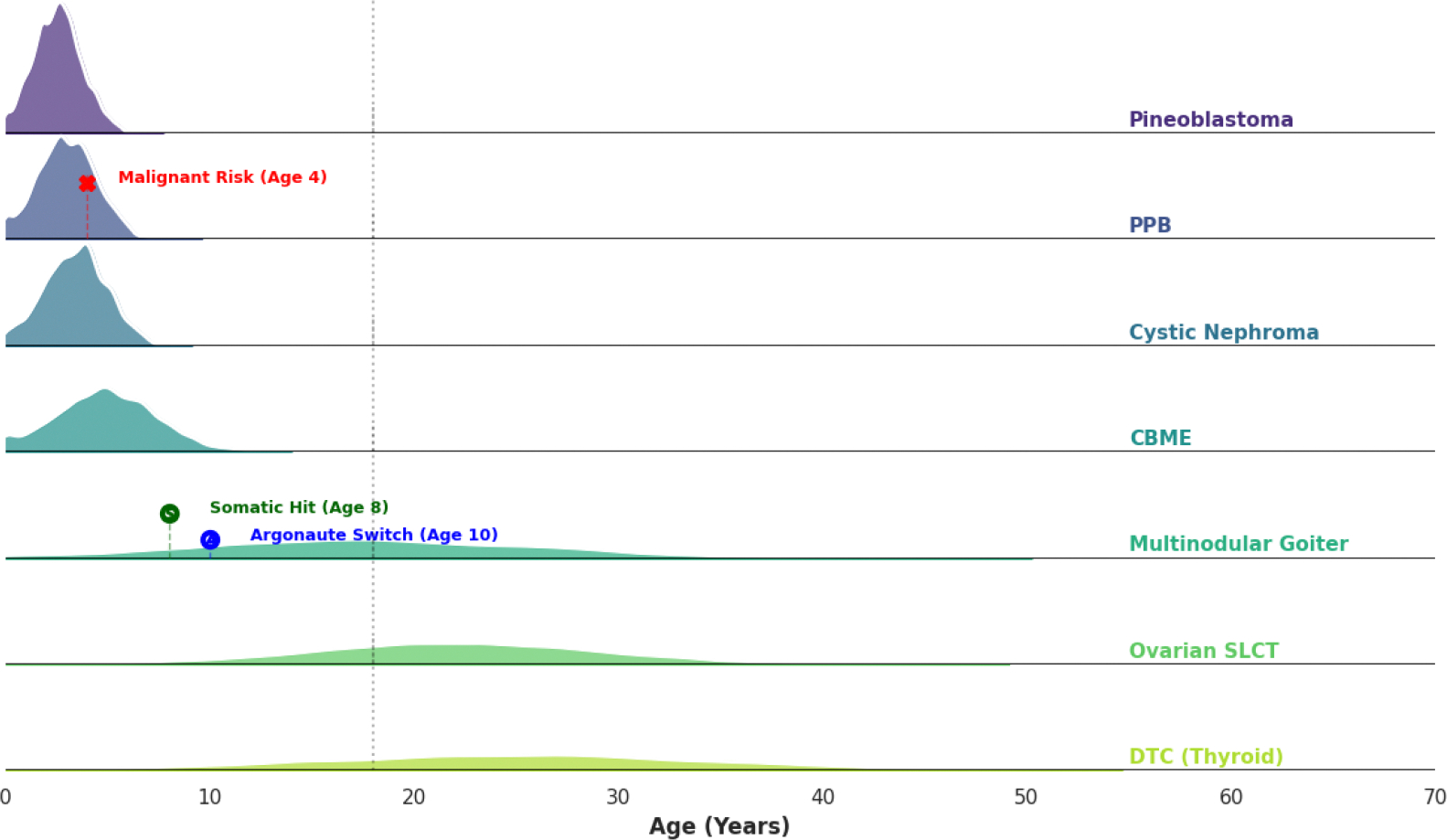
Chronological Distribution of DICER1-Associated Lesions. Graphical representation of the “natural history clock” of the syndrome. Note the high-intensity peak for pleuropulmonary blastoma (PPB) and cystic nephroma (CN) within the first 5–8 years of life, followed by the secondary emergence of thyroid multinodular goiter (MNG) and ovarian Sertoli-Leydig cell tumors (SLCT) in the second and third decades. CBME, ciliary body medulloepithelioma; DTC, differentiated thyroid carcinoma.

Like pulmonary developments, cystic nephroma (CN) usually manifests as a multilocular renal mass between ages 1 and 4 [20, 21, 22]. Distinguishing CN from Wilms tumor is vital because the two are often radiographically indistinguishable yet require opposite management [20]. Whereas Wilms tumor necessitates aggressive neoadjuvant chemotherapy and often radical nephrectomy, DICER1-related CN follows a unique etiological pathway favoring cystic dysplasia that is inherently chemotherapy-resistant [20, 21, 22]. Recognizing this allows for nephron-sparing surgery, preserving renal function, and avoiding the toxicity of ineffective systemic treatments [22]. The diagnosis is pathologically confirmed by the presence of thin fibrous septa and a characteristic lining of hobnail epithelium. While these features signify a benign lesion, the resulting cysts can still cause significant mass effects [22]. Genomic characterization has further differentiated these lesions into specific molecular classes, reinforcing that DICER1-associated renal tumors exist on a biological spectrum ranging from benign CN to more aggressive renal sarcomas, such as anaplastic sarcoma of the kidney [22, 23, 24]. The distinct temporal window of these early childhood manifestations compared to later adolescent risks is visualized in Figure 1.

#### Thyroid and Ovarian Patterns in Adolescence and Adulthood

As patients transition into adolescence, the phenotypic spectrum shifts toward the thyroid and reproductive organs [1, 25]. Multinodular goiter (MNG) is the most prevalent manifestation, often appearing as bilateral and multifocal hyperplastic nodules [26, 27]. While most of these nodules remain indolent, family-based cohort studies show an elevated risk for thyroid malignancies [28]. Patients require vigilant monitoring to detect rare shifts toward differentiated thyroid carcinoma or the more aggressive thyroblastoma, a primitive multilineage thyroid neoplasm characterized by fetal morphology [1, 25, 29, 30, 31]. Recent cytopathological studies emphasize that DICER1-related thyroid nodules may show distinct features, such as specialized follicular patterns and the absence of classic papillary thyroid carcinoma markers, which assists in their differentiation during fine-needle aspiration [27, 29, 32]. Clinical and molecular characteristics often alter treatment strategies for these thyroid malignancies, moving away from aggressive surgery when indolent features are present [33, 34, 35].

Ovarian Sertoli-Leydig cell tumors (SLCT) represent another critical facet of the syndrome’s course, typically diagnosed before age 40 [10, 36]. These tumors are frequently hormonally active; the resulting androgen excess often leads to virilization, which can serve as a diagnostic trigger for genetic testing [36]. Recent research has identified that intronic germline DICER1 variants may also predispose patients to SLCT, necessitating comprehensive sequencing [37]. Immunohistochemical analysis has demonstrated that the second hit typically occurs within the Sertoli cell component [10]. The presence of heterologous elements, such as gastrointestinal-type epithelium or skeletal muscle, further characterizes DICER1-associated SLCTs and helps distinguish them from other sex cord-stromal neoplasms [36]. Early detection is essential, as localized disease can often be managed with fertility-sparing surgery alone [1, 36]. Beyond SLCT, DICER1 mutations are also found in Müllerian adenosarcomas and rare primary intracranial spindle cell sarcomas with rhabdomyosarcoma-like features [38, 39]. The emergence of these rarer, extra-gonadal manifestations underscores the pleiotropic nature of the syndrome and highlights the necessity for multidisciplinary awareness to ensure early diagnostic intervention across diverse organ systems.

### Basic Molecular Concepts: The Drivers of Pathogenesis

#### The miRNA-140/FGF9 Signaling Axis

The molecular pathogenesis of early DICER1 lesions, particularly in the lung, is rooted in the disruption of paracrine signaling between the epithelium and the mesenchyme [16]. Under normal conditions, epithelial DICER1 processes miRNAs that act as a regulatory brake on Fibroblast Growth Factor 9 (FGF9) expression [16]. When DICER1 is deficient, the loss of these regulatory microRNAs, specifically miR-140, leads to the uncontrolled overexpression and secretion of FGF9 [16]. This excess signaling forces mesenchymal cells to remain in a state of primitive proliferation rather than differentiating into mature alveolar structures [6, 16]. This mechanism explains why Type I PPB begins as a multicystic dysplasia driven by signaling errors rather than immediate genomic instability [11, 16].

#### The let-7 Feedback Loop and Oncogenic Unleashing

Beyond FGF9 signaling, cellular homeostasis is compromised by the disruption of vital negative feedback loops. Research has identified that let-7 microRNAs regulate DICER1 expression itself, creating a feedback mechanism that ensures precise control over miRNA biogenesis [40]. In DICER1-deficient cells, the drop in mature let-7 levels results in the functional unleashing of oncogenic networks that govern cellular proliferation and stemness [2, 40]. This molecular environment promotes a dedifferentiated state, providing the biological foundation for the expansion observed as benign cysts progress into solid, high-grade sarcomas [2, 23, 41].

#### The Two-Hit Hypothesis and Neomorphic 3p-Strand Bias

**Figure 2:**
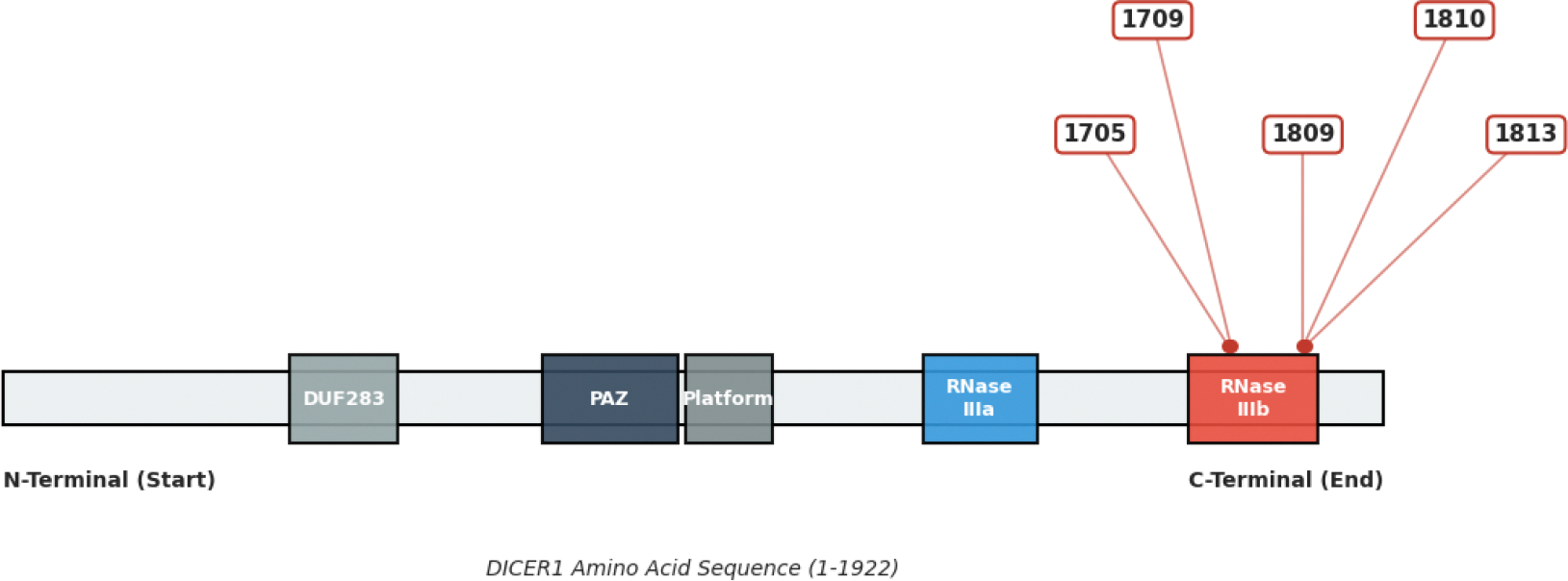
Somatic RNase IIIb Hotspot Mutation Architecture of the DICER1 Protein. A domain map highlighting the concentration of metal-binding somatic mutations (red) clustered specifically within the critical RNase IIIb catalytic hotspot (red box). Each number points to a specific codon identified (1705, 1709, 1809, 1810, 1813) that is mutated in high-grade sarcomas.

Progression to malignancy typically follows the two-hit model: a germline loss-of-function mutation followed by a somatic missense variant localized to hotspot codons (E1705, D1709, G1809, D1810, and E1813) within the RNase IIIb domain [2, 10, 12] (Figure 2).

**Figure 3:**
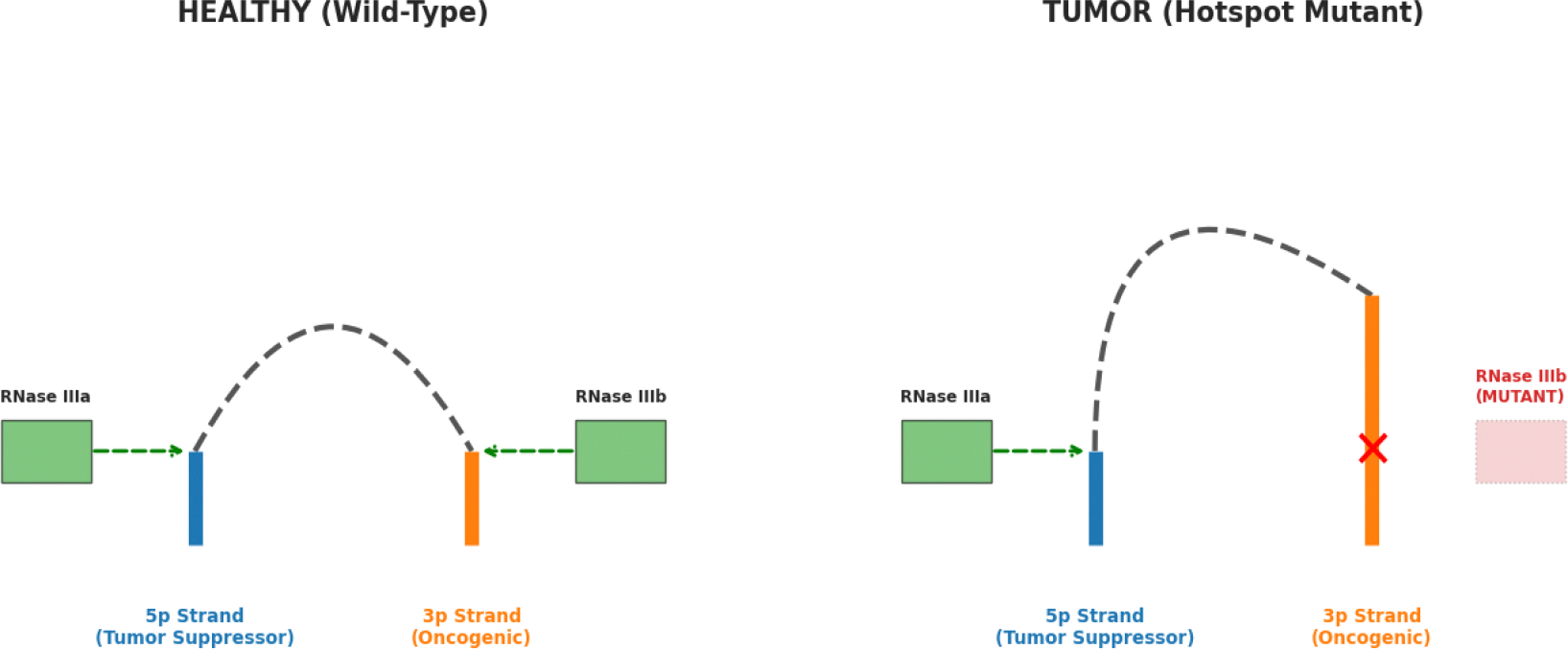
Molecular Mechanism of the Neomorphic 3p-Strand Bias. Left figure: Wild-type (healthy) DICER1 processing, showing symmetrical cleavage of 5p and 3p miRNA arms. Right figure: The “Hotspot” mutant mechanism in tumor, where loss of RNase IIIb activity results in failed 5p cleavage while maintaining 3p processing. This “Argonaute strand switch” leads to the depletion of 5p tumor suppressors and the oncogenic flooding of 3p-derived miRNAs.

Recent structural analysis shows that missense variants in the platform domain can also inhibit miRNA biogenesis and lead to tumor susceptibility [9]. When RNase IIIb hotspot mutations occur, the enzyme acquires a neomorphic gain-of-function activity [42]. The mutated DICER1 remains capable of cleaving the 3p-arm of the pre-miRNA via the intact RNase IIIa domain but fails to cleave the 5p-arm [9, 42]. This results in a profound miRNA strand bias, recently described as an argonaute strand switch, where the cell is depleted of 5p-derived tumor suppressors and flooded with an overabundance of 3p-strands that function as oncogenic microRNAs to aggressively rewire growth pathways [42]. This biochemical failure and the resulting shift in the miRNA landscape are illustrated in the comparative molecular models in Figure 3.

### Identification of Predictive Markers for Malignancy

Clinically, the most significant indicator of malignancy is the rate of volumetric growth; a previously stable mass that begins to expand rapidly is a definitive red flag [1, 11]. Radiologically, the hallmark of the malignant shift is the solidification of cystic spaces, including the appearance of internal mural nodules or thickened, enhancing septations [1, 11, 43]. Pathologically, the transition is marked by increased mitotic activity, cellular atypia, and the overgrowth of the primitive stromal component [15, 16]. On a molecular level, the detection of somatic RNase IIIb hotspot mutations provides definitive evidence of the second hit [10, 42, 41]. High-sensitivity detection methods, such as drop-off droplet digital PCR, have been developed to identify these hotspot mutations even in low-cellularity samples [13]. Furthermore, the presence of co-mutations in TP53 and KRAS serves as a marker for particularly aggressive primary DICER1-sarcomas [44]. Emerging research into non-invasive molecular markers, such as circulating tumor DNA (ctDNA) and concurrent DNA and RNA sequencing, represents the next frontier in monitoring [13, 45, 46].

### Surveillance and Management Strategies

The 2024 consensus guidelines advocate for a risk-stratified surveillance approach tailored to specific chronological windows of vulnerability [1] (Table 1). For pulmonary health, children should undergo regular chest X-rays every six months until age eight, supplemented by baseline low-dose CT scans to detect occult Type I PPB [1, 43]. Renal surveillance follows a parallel timeline, utilizing abdominal ultrasound every six months until age eight to identify cystic nephroma at a treatable stage [1, 21, 22, 24]. These intervals are strategically timed to coincide with the peak incidence periods for early childhood manifestations [1, 11]. Management of the reproductive and thyroid organs requires a more prolonged monitoring strategy. For females, pelvic ultrasound is now recommended every six months starting at birth or upon genetic diagnosis to monitor for ovarian SLCT; this immediate start is critical as approximately 15% of these tumors occur before the age of eight [1, 36]. Thyroid surveillance typically begins at age eight with a baseline ultrasound, followed by periodic monitoring to distinguish prevalent benign multinodular goiter from rare progression to differentiated thyroid carcinoma or thyroblastoma [1, 25, 27, 28, 29, 34, 35]. Furthermore, as the number of identified carriers grows, clinicians must carefully evaluate variants of uncertain significance. Utilizing updated structural data and variant curation guidelines is essential to prevent both the risks of under-surveillance and the morbidity of unnecessary surgical intervention [5, 9, 47]. A consolidated summary of the 2024 risk-stratified surveillance modalities and frequencies is provided in Table 1. Clinicians must also remain aware of intraocular medulloepitheliomas, which can occur as part of the syndrome [48, 49, 50]. Ultimately, integrating these diverse clinical findings into a centralized monitoring plan allows for the early detection of localized disease while maintaining the high specificity required to avoid the morbidity of over-treatment in germline carriers.

## Conclusions

The clinical course of DICER1-associated lesions is defined by a constant biological tension between indolent developmental dysplasia and aggressive sarcomatous transformation [2, 11]. While most of these lesions follow a benign or even regressive path, their potential for sudden, rapid malignancy necessitates a strategy of lifelong, evidence-based vigilance. Ultimately, our success in managing these patients is becoming increasingly tied to our understanding of the molecular switch, specifically the neomorphic 3p-strand bias and the argonaute strand switch, that triggers the transition from a stable cyst to a high-grade neoplasm [2, 42].

**Figure 4:**
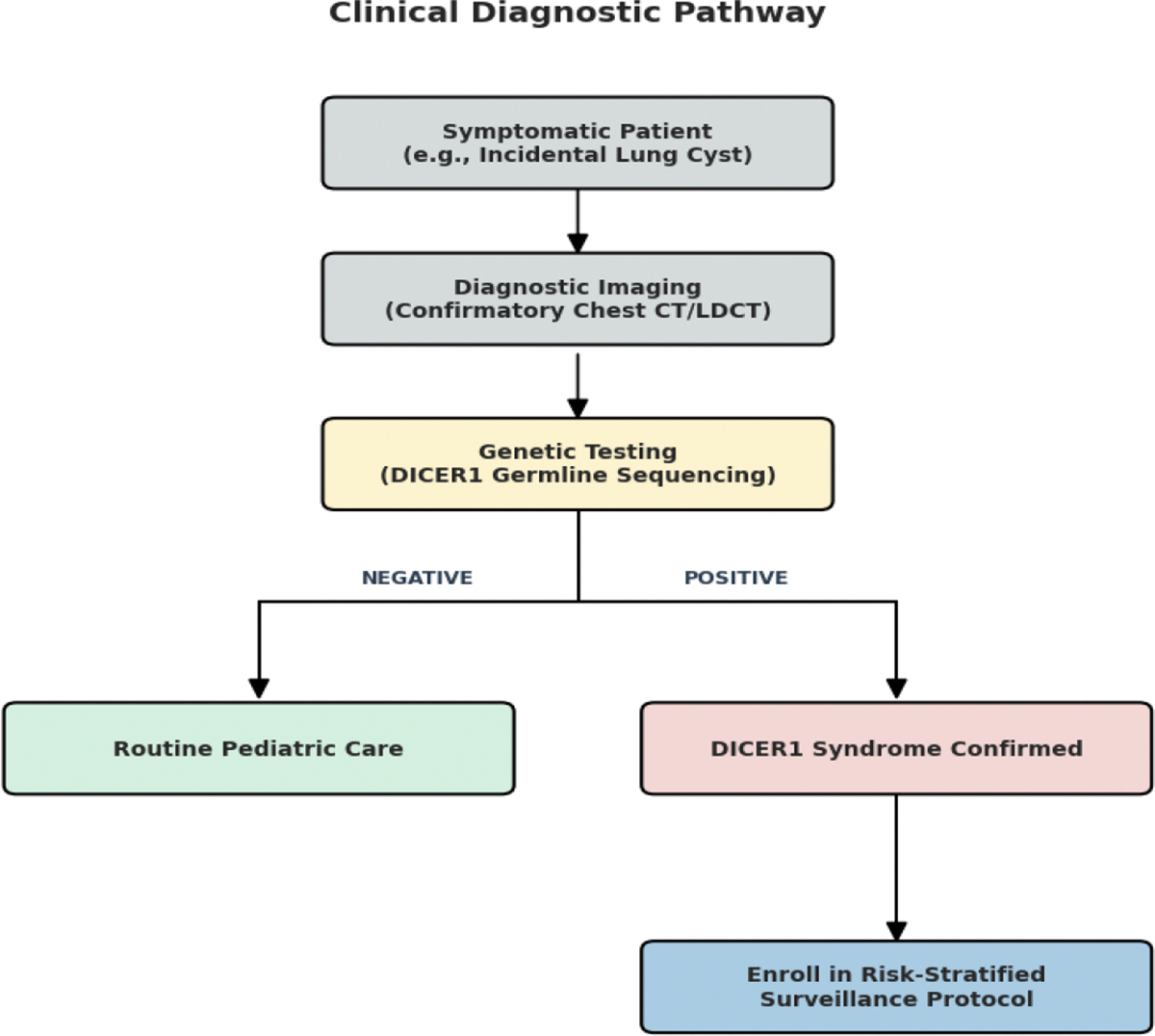
Clinical Diagnostic Algorithm for Suspected DICER1 Syndrome. A decision-tree framework for clinicians managing incidental findings, such as pulmonary cysts. The pathway illustrates the integration of diagnostic imaging and germline sequencing to differentiate routine pediatric cases from confirmed *DICER1* syndrome requiring longitudinal management.

Looking ahead, the integration of the 2024 surveillance standards with high-sensitivity tools like droplet digital PCR for ctDNA and comprehensive RNA sequencing represents the next major frontier [1, 13, 45, 46]. However, as the volume of screening data grows, so does the burden of accurately classifying these early-stage lesions. Emerging computational research, such as the use of Vision Transformer models, offers a promising way to address this by identifying subtle imaging patterns in tumors like PPB that might be overlooked during a standard review [51]. To assist in the practical application of these findings, Figure 4 outlines the diagnostic pathway from initial symptomatic presentation to genetic confirmation and enrollment in surveillance.

By combining these types of predictive tools with the deep longitudinal data held by the International DICER1 Registry, we can maximize the potential for definitive cures while minimizing the long-term clinical burden on this patient population [1, 22, 51]. As our understanding of the neomorphic strand-switch and emerging radiographic biomarkers matures, the focus of *DICER1* care will continue to shift toward highly individualized, evidence-based interventions that define the next frontier of pediatric and adolescent oncology.

## Figures and Tables

**Table 1:** 2024 Evidence-Based Summary of DICER1 Surveillance Guidelines/Recommendations. A clinical reference table summarizing the recommended screening protocols for the primary manifestations discussed in this text. Guidelines prioritize semi-annual imaging for early-childhood lung and kidney risks, transitioning to annual thyroid and ovarian monitoring in older cohorts.

Organ System	Screening Modality	Age Range	Frequency
Lung (PPB)	Chest X-ray / LDCT	Birth - 8 years	Every 6 months
Kidney (CN)	Abdominal Ultrasound	Birth - 8 years	Every 6 months
Thyroid (MNG/DTC)	Physical Exam / US	Start at 8 years	Annual
Ovaries (SLCT)	Pelvic Ultrasound	Birth - 40 years	Annual

## Key Points

- DICER1 syndrome is an autosomal dominant disorder caused by germline mutations that disrupt microRNA biogenesis.
- The syndrome follows a distinct chronological clock, beginning with pulmonary and renal lesions in early childhood.
- Pleuropulmonary blastoma (PPB) is a critical sentinel event, often starting as a multicystic Type I lesion.
- Cystic nephroma (CN) in DICER1 patients is chemotherapy-resistant and requires nephron-sparing surgical approaches.
- In adolescence and adulthood, the focus shifts toward thyroid nodules (MNG) and ovarian Sertoli-Leydig cell tumors (SLCT).
- Virilization in females due to androgen excess is a significant indicator of an underlying DICER1-related ovarian tumor.
- Malignant transformation typically requires a somatic “second hit” within the RNase IIIb domain.
- The neomorphic argonaute strand switch causes an oncogenic overabundance of 3p-miRNA strands.
- Rapid volumetric growth and cystic solidification on imaging are primary indicators of a malignant shift.
- Modern management integrates 2024 surveillance protocols with high-sensitivity molecular tools like ctDNA and RNA sequencing.
